# Relationships between Cornell Musculoskeletal Discomfort Questionnaire and Online Rapid Office Strain Assessment Questionnaire

**Published:** 2018-11

**Authors:** Ardalan SHARIAT, Joshua A. CLELAND, Mahmoud DANAEE, Mehdi KARGARFARD, Vahide MORADI, Shamsul BAHRI MOHD TAMRIN

**Affiliations:** 1. Sports Medicine Research Center, Neuroscience Institute, Tehran University of Medical Sciences, Tehran, Iran; 2. Dep. of Occupational Health, Faculty of Medicine and Health Sciences, University Putra Malaysia, Selangor, Malaysia; 3. Dept. of Physical Therapy, Franklin Pierce University, Manchester, New Hampshire, USA; 4. Academic Enhancement and Leadership Development Center (ADeC), High Impact Research Building, University of Malaya, Kuala Lumpur, Malaysia; 5. Dept. of Exercise Physiology, Faculty of Sport Sciences, University of Isfahan, Isfahan, Iran; 6. Dept. of Orthotics and Prosthetics, Faculty of Rehabilitation, Iran University of Medical Sciences, Tehran, Iran

**Keywords:** ROSA, Computer, Feedback, Worker assessment, Checklist

## Abstract

**Background:**

The Rapid Office Strain Assessment (ROSA) is a tool employed online to screen office workstations, which may require modification to decrease musculoskeletal discomfort of workers. This study aimed to examine if the ROSA is able to evaluate pain severity in the lower back, shoulder and neck of office workers accurately.

**Methods:**

Overall, 142 participants (height: 1.80 ± 0.15 m, BMI: 26.08± 6.70, age: 35±15 yr) with at least a year of working experience completed both questionnaires, the online ROSA and the Cornell musculoskeletal discomfort, in 2016 in Malaysia.

**Results:**

Relationship between the total scores of both questionnaires for lower back, shoulder and neck pain were significant but exhibited a weak to moderate relationship (range of *r* values from 0.012 (CI 95%, −0.153–0.176) to 0.503 (CI 95%, 0.369–0.616).

**Conclusion:**

The online ROSA does not appear to be a reasonable tool for evaluating the severity of lower back, shoulder and neck pain among office workers as the correlations were low. We suggest continued use of the musculoskeletal discomfort questionnaire. Additional studies are required to further examine the ROSA for other anatomical regions.

## Introduction

Persistent non-neutral positions of the upper limbs including static and sustained periods in front of the computer, uncomfortable postures of the shoulders, upper back and neck are among numerous risk factors related to musculoskeletal conditions at the workplace (1,2).

Workplace musculoskeletal disorders are the cause of a considerable amount of sickness related absence from work, which is higher than any other health disorder and accounts for roughly a half of all disorders at workplace in members of European Union. Nearly a quarter of European workers state they experience muscular pain in their upper limbs, shoulders and neck (3). This issue related to the computer use in offices had been associated with back segments, upper limbs and neck (4,5). Sit-stand, or having some active breaks would show a possibly useful response of decreased lumbar flexion which might have the potential to avoid musculoskeletal conditions (6).

For example, more than three out of five office employees in Canada depend on computers to do their occupation and the annual rate of musculo-skeletal conditions are mainly influenced by the above-mentioned risk factors (7). Therefore ergonomic evaluations and training have proactively decreased these factors in the workplace (8,9). Primary risk factors are examined with screening tools including the RULA (rapid upper limb assessment) and REBA (rapid entire body assessment) are regularly used for ergonomic assessment recommendations. However, these tools succeed in assessing particular risk factors as their constituents tend to be all-purpose in nature to accommodate a range of jobs (10). Furthermore, it has yet to be identified if the gaps in the levels of action of these posture assessment tools are able to be used in tasks reliant on computers and often the validity is unidentified (11).

The Rapid Office Strain Assessment (ROSA) pen-and-paper checklist is an example of a risk factor screening tool intended to recognize the necessity for on the job intervention at an office workplace (12). Using the CSA (Canadian Standards Association) standards for Office Ergonomics (CSAZ412) as its foundation, the ROSA function is to detect academically-classified MSD risk factors (13). Consequently, subsections including telephone, mouse, monitor and keyboard in addition to chair are employed to integrate the above-mentioned risk factors into the tool (14). Ultimate score of ROSA offers an overall image of risks of musculoskeletal discomfort achieved by adding the accumulated scores of each subgroup. A significant connection linking musculoskeletal discomfort with final ROSA score (12). Office workstations self-assessments using the present ROSA online application demonstrate promise in terms of supporting workers to reduce risk factors connected to musculoskeletal discomforts and reduce levels of discomfort (15).

Elevated levels of musculoskeletal discomfort among workers have been connected to ROSA scores of 5 or above (16). If this occurs the ergonomic consultants’ services are required to perform the screening evaluations which requires increased expenditures from the organization and investigators are confronted with hurdles when employing the ROSA (16). In this setting, when a workplace is entirely dependent on proficient advisors for information concerning changes to any workstation it will undergo financial and time constraints regardless of the fact that a strong point of ROSA is effective screening (1,7).

There are various kinds of questionnaire to determine the musculoskeletal discomfort experienced by office workers. The Nordic (17) and Cornell (18) are the most common questionnaires used for this purpose. The Cornell questionnaire was used to determine the level of pain among office employees in response to rest breaks and ergonomic modifications. Musculoskeletal discomforts, particularly pain severity among office employees, can be collected by the Cornell questionnaire shown to be a valid and reliable tool (19). Nonetheless, since the ability of ROSA to detect the discomfort severity among employees has not been authenticated there are concerns that the Cornell scores’ results may differ from the ROSA. Consequently, the novelty of the study is in the population being studied and the outcome relationships of the ROSA and Cornell total score in the neck, shoulders and lower back. Thus, the key goal of this study was to evaluate the online ROSA questionnaire’s validity regarding the magnitude of lower back, shoulder and neck discomfort among employees of the office. There would be a significant and positive relationship between the ROSA total discomfort score in the neck, shoulder, lower back and Cornell total scores.

## Materials and Methods

### Subjects

This study selected a Malaysian government office as the site of data collection. The workers who work up to 8 h a day with a computer were chosen as the study population. For screening, this study used the Cornell questionnaire and 142 participants, who at least reported one case of critical pain in the lower back, shoulders and/or neck, were randomly selected, with a Random Number Table, from a total 752 subjects in 2016. To be involved in this study subjects had to be able to understand the questionnaire’s content, be between the age of 20 to 50 yr and without any physical disorders, which would influence their ability to do basic physical activity. Before they were included in the study they were asked to sign the informed consent, and guidelines of the 1964 Helsinki declaration regarding all processes involving human subjects.

This study was approved by Ethic Committee at the University Putra Malaysia (UPM) (FPSK - EXP16-P046). The Clinical Trial ID for this study is NCT02874950.

### ROSA online questionnaire

Comparable risk factor identification information of the original ROSA is also seen in the ROSA online version. However, modifications were made to the online version was subsections related to the chair, monitor, telephone, mouse, and keyboard to enable researchers to utilize similar risk factor diagrams in the assessment process. Consequently, the general figures of the two key groups of the online ROSA tallied by the software were lower than 5 and higher than 5 (12). The color red, representing risk, was utilized to show scores 5 and more.

### Concurrent validity

Concurrent validity of the online ROSA questionnaire was examined. To do so the subjects were first requested to complete the Cornell musculoskeletal discomforts questionnaire. They were given three separate items in the Cornell questionnaire: 1). Throughout the last work week how often did you undergo discomfort, pain or ache (is shown by D), 2) If you endure discomfort, pain, or ache, how painful was this? (is shown by E), 3) If you experienced discomfort, pain, or ache, did this affect your capability to work? (is illustrated by I). Those participants who stated discomfort or pain in the Cornell questionnaire might also report the same in the online ROSA, and similarly, those who did not report discomfort or pain in the Cornell questionnaire would be expected to report “Never” in their answers in the ROSA.

### Statistical analysis

Before data analysis was performed the normality of data was estimated according to kurtosis and skewness and findings revealed that the data were normally distributed. To determine the relationship of levels of discomfort in the lower back, shoulders and neck between ROSA total scores and the Cornell questionnaire, the Pearson Product Moment Correlation was employed. Using SPSS version 23.0 data were analyzed. *P*<0.05 was considered a significant correlation. The distribution pattern of scores in the domains was identified using the skewness and kurtosis analysis.

## Results

### Demographics

Overall, 142 subjects completed the questionnaire including 49 (34.5%) male 93 and (65.5%) female. They were strictly comprised of Malaysian citizens (aged 35±15 yr, with the body mass of 26.08± 6.70, and height of 1.80 ± 0.15 m; [mean ± SD]) they spent at least 8 h in front of a computer each day and with at least 1-year working experience. There were less than 2% of missing values and no systematic pattern was identified as in data screening no out-of-range cases were detected.

### Concurrent validity

#### Relationships between total scores of discomfort in neck

ROSA total scores and Cornell Neck D, Neck E and neck total scores had a weak significant relationship (Table 1). No-significant correlation was found between Neck I and ROSA total scores.

**Table 1:** Correlation coefficients between ROSA total score of discomfort in the neck, shoulder, lower back and Cornell total scores

**Neck**				
***Variable***	***D***	***E***	***I***	***Total***
r	.367^**^	.259^**^	−0.077	.385^**^
*P*-value	0.001	0.002	0.361	0.001
Right Shoulder				
r	.485^**^	−0.058	0.014	.340^**^
*P*-value	0	0.492	0.872	0
Left Shoulder				
r	.503^**^	−0.058	−0.058	.299^**^
*P*-value	0	0.489	0.495	0
Lower back				
r	.390^**^	.012	−.075	.274^**^
*P*-value	.000	.887	.374	.001

** Significant at the 0.01 level (2-tailed)

#### Relationships between total scores of discomfort in shoulder

ROSA total scores and Shoulder R.D and Shoulder R total scores had a weak significant relationship (Table 1). No significant relationships were found between Shoulder R.I and Shoulder R.E and ROSA total scores. A weak significant relationship was found between ROSA total scores and Shoulder L.D and Shoulder L total (Table 1). No significant relationship was seen between Shoulder L.I and Shoulder L.E and ROSA total scores.

#### Relationships between total scores of discomfort in lower back

Weak significant relationships were observed between ROSA total scores and Lower back D and Lower back total (Table 1). Nevertheless, there was not any significant relationships between ROSA total scores and Lower back E and Lower back I.

No significant correlations were found between weights and, ROSA total scores, Shoulder R total, Neck total, lower back total and Shoulder L total (Table 2).

**Table 2:** Correlation coefficients between the total score of discomfort in lower back, neck shoulder in Cornell questionnaire and ROSA total scores and Weight

	***ROSA total***	***Neck total***	***Shoulder R total***	***Shoulder L total***	***Lower back total***
r	.114	.034	−.034	−.105	.077
*P* value	.177	.690	.687	.214	.360

## Discussion

This study aimed to determine the validity of online ROSA questionnaire to evaluate pain severity in the lower back, shoulder and neck among office employees. According to the findings of all subsections and final scores, worker and observer scores had significant correlations. Moreover, ROSA scores were significantly but not highly related to those of discomfort and thus exhibits low validity. A significant relationship was found between discomfort and ROSA scores. These results are comparable in magnitude to (12) with the magnitude of the link between ROSA final score and whole body discomfort which ranged from *r*= 0.40 to *r*= 0.70. The findings of this study are in line with a previous study that showed a significant link between the posture of sitting and working with musculoskeletal discomforts (20).

To evaluate concurrent validity the Cornell musculoskeletal discomfort questionnaire was also employed. The studies of musculoskeletal disorders consider International Musculoskeletal Disorder (MSD) Questionnaire as an essential feature (21). Since the CMDQ examines discomfort levels according to frequency and severity and it also analyzes the rate at which work performances of individual are negatively influenced and this statement was mentioned by previous recent studies (22,23). Concerning this study’s objective, only severity and total scores were emphasized as well as discomfort in the lower back, shoulders and neck because of a high amount of reports stating pain in these regions (69.7%) (11). Hedge et al. was employed to help the Cornell questionnaire’s scoring (24).

The present research can be distinguished from earlier studies in that there was no correlation in discomfort scores and total body discomfort scores tended to have greater correlations with ROSA scores. Lumbar disc herniation among office employees occurs due to the risk factor of (25) prolonged sitting on a daily basis (26). Maintaining a posture of sitting during long hours in static postures may cause alterations in muscular activity of the cervical spine and shoulder stabilizers (27). This continuous activity may lead to muscle fatigue and result in WRMSD. This may lead to sciatica, a disorder of the sciatic nerve resulting in sharp pain down the (26). The posture of sitting at the computer resulted in workers adapting throughout the workday and interaction with the other features of the workplace leads to muscle tension in the shoulder and neck (14).

Thus analyses must also include leg discomfort, not only because an estimated 23% of all office employers undergo sciatica (28), but also because it is a consequence of stated pain from damage to the lower back area (29). Consequently, these factors may be accountable for distinctions in the connection between uneasiness and ROSA scores in both earlier and the present study on the ROSA. A traditional paper version of the discomfort questionnaire was created while monitoring a group of employees. The way in which employees stated discomfort may have been influenced by feedback on the evaluation since this research received negative feedback.

Preferably, recommendation for doing things in different ways in the future, as well as suggesting comments to employees when they have scored their assessment incorrectly are of the important aims of feedback. Any differences in employee assessment scores were directly considered as false replies since the assessments of trained observers were very important in this research. Additionally, there is also the likelihood that employees failed to understand their mistakes because of the amount of provided feedback. The findings of ROSA does not relate directly to the bad equipment in the workplace reported by the workers, but rather with the improvement of workers' posture, optimizing its use (14) and findings can support the results of this study.

The online ROSA’s sensory aspect might have caused greater correlations if it was qualitatively considered. The assessment aspect revealed the maximum correlation with the numeric scale. This was not an unpredicted outcome since the online ROSA asks the participants to judge their pain based on the intensity. The online ROSA questionnaire’s main purpose is to measure the evaluative aspect of pain. The effective and sensory sub-scales are satisfactory but it cannot guess the pain severity or score in particular areas. Based on the online ROSA questionnaire, the evaluative side is recognized as a worldwide outcome of the subjective comprehension of associated pain (cognitive dimensions of pain), but since this is merely a sub-scale which includes a simple decision of pain intensity, it oversimplifies the description. The online ROSA presented worthy office ergonomic metric belongings. By representing employers’ insights of their signs and symptoms together with the influences of those symptoms on their behaviors, the online ROSA can offer significant data to a physician and increase clinical results to design treatment.

There are still unilateral postures such as when workers hold the phone between their head and shoulder which cause muscle fatigue and decreased flexibility due to the tension that prevents the muscles from working on his greatest performance. Therefore, work-related gym program development adoption concentrated on exercises which have the aim of relaxing the musculature of the neck and shoulders segments may reduce fatigue and, therefore, lessen pain and improve flexibility.

## Conclusion

The Cornell questionnaire cannot be employed as a tool to estimate the total level of discomfort in the lower back, shoulders, and neck among office employers. Additional research is needed using this tool, specifically concerning its ability to assess pain in other body parts and its harmony with other questionnaires related to musculoskeletal discomfort-, including the Nordic questionnaire.

## Ethical considerations

Ethical issues (Including plagiarism, informed consent, misconduct, data fabrication and/or falsification, double publication and/or submission, redundancy, etc.) have been completely observed by the authors.
